# Equivalence and noninferiority trials – are they viable alternatives for registration of new drugs? (III)

**DOI:** 10.1186/1468-6708-5-8

**Published:** 2004-08-17

**Authors:** Cornel Pater

**Affiliations:** 1 Hannover, Germany

## Abstract

The scientific community's reliance on active-controlled trials is steadily increasing, as widespread agreement emerges concerning the role of these trials as viable alternatives to placebo trials. These trials present substantial challenges with regard to design and interpretation as their complexity increases, and the potential need for larger sample sizes impacts the cost and time variables of the drug development process. The potential efficacy and safety benefits derived from these trials may never be demonstrated by other methods. Active-controlled trials can develop valuable data to inform both prescribers and patients about the dose- and time-dependent actions of any new drug and can contribute to the management and communication of risks associated with the relevant therapeutic products.

## Background

In an era of cost containment, the need for rigorous examination of the cost-effectiveness of drugs, as well as their clinical effectiveness, is widely recognized not only by governments but also by the pharmaceutical industry [1-4]. Messages framed differently, but with the same basic content, have reached the community of prescribing physicians, who have come to understand that, although an effective drug may be prescribed for patients who would benefit from it, unless the drug is cost-effective, the resources that are expended might produce greater benefits for other patients. Such messages and updated recommendations to prescribing doctors, in addition to results derived from recent large, randomized trials, continue to have only minimal, if any, impact on the prescribing habits of doctors.

The latest such example [5] concerns the outcomes of the Antihypertensive and Lipid-Lowering Treatment to Prevent Heart Attack Trial (ALLHAT), indicating that diuretics could be more effective than angiotensin-converting enzyme (ACE) inhibitors or calcium channel blockers in the treatment of hypertension, and at a much lower cost [6]. Despite this convincing evidence, a study presented at the annual conference of the American Heart Association in March 2004 showed that spending on antihypertensive drugs essentially doubled (from approximately $6 billion to approximately $12 billion) between 1990 and 2002. The explanation most commonly offered is that "doctors selected the more costly antihypertensive agents."

Since cost-effectiveness is conventionally required for evaluating the efficacy of alternative healthcare interventions, the perspective commonly taken is that of the health services [7]. Therefore, establishing the superiority or equivalence of a new intervention relative to the standard one has been extended not only to new drug entities, but also to generic versions of innovator drugs [8], surgical techniques [9], medical devices [10], and such diverse factors as medical protocols [11].

Cost-effectiveness and clinical effectiveness should be pursued simultaneously to ensure that health care is efficient, ethical, and beneficial to patients. This paper deals with only one aspect of clinical effectiveness: drug treatment benefit and how it may be ascertained from claims of therapeutic equivalence.

## Ethical Issues

The basis for the scientific and ethical underpinnings for the design and conduct of randomized clinical trials is the *uncertainty principle*, which states that a patient should be enrolled in a randomized controlled trial only when substantial uncertainty exists as to which of the trial treatments would benefit the patient more [12]. From this principle derives the fundamental ethical challenge of equivalence trials, reflected in the researcher's explicitly expressed belief that "the new drug might be not different from the old drug," a fact that should be acknowledged in the consent process whereby trial subjects are informed that "it is not known which drug is better or whether they are the same."

Nevertheless, demonstrating equivalence of the drugs being compared implies starting from the assumption that the new drug is better. In other words, the hypothesis to be tested in equivalence trials (and the hypothesis that is refuted if equivalence is shown) is that one treatment is superior to the other. Altruistic patients are more likely to agree to participate in such a trial, whereas other, less altruistic patients are more likely to decline participation, as their interest lies in treatments with proven efficacy. Obviously, this situation is more patient-favorable than are placebo-controlled trials, in which the individual patient's well-being may be subordinated to the good of others [13,14]. Placebo-controlled trials are still used extensively to demonstrate the effectiveness of new drugs; however, a paradigm shift appears to be steadily emerging in this area [15,16]. Speaking metaphorically, Urquhart stated that placebos are predestined to be "roadkill on the highway of medical progress" [17].

For circumstances in which no increased risk for patients is foreseen, use of placebo-controlled trials seems appropriate and ethical, provided the patients are fully informed and that they give their written, informed consent. However, if these patients and their doctors were to find the placebo-controlled studies inappropriate, and if they were to exercise their option in large numbers, these studies would become unfeasible, regardless of the ethical justifications, scientific considerations, views of the trial sponsor, or, ultimately, the expectations of regulatory authorities.

Apart from the extreme opinions that challenge the placebo-controlled trials as unethical [18-24] and those that advocate proactive use of the active-controlled equivalence trials [25,26,31] or question their scientific merits [16,26-32], a balanced approach is needed (i.e., one that recognizes the use of placebos in instances wherein efficacy cannot otherwise be demonstrated, and the use of active-controlled trials as the design of first choice when scientifically sound circumstances require it). Simultaneous use of both alternatives might be necessary in selected cases.

## The issue of *assay sensitivity*

Defined as "the ability of a study to distinguish between active and inactive treatment," assay sensitivity is the *sine qua non *for the validity of equivalence claims derived from any active-controlled equivalence/noninferiority study. Methodological flaws affecting one or several of the specific elements inherent in assay sensitivity itself seem to have been more a rule than an exception in many trials carried out during the past decade. Illustrating this point is a systematic review of trials published between 1992 and 1996 that claim equivalence [33]. In the review, the authors showed that:

• 88 papers were evaluated for five equivalence-specific methodological attributes.

• Only 45 (51%) of the 88 reports specifically identified demonstration of equivalence as their aim; the others attempted to show superiority or did not state any research aim.

• An equivalence boundary was set and confirmed with an appropriate statistical test in 23% of the reports; in 67% of reports, equivalence was declared after a failed test for comparative superiority; in 10%, the claim of equivalence was not evaluated statistically.

• Sample sizes were calculated in advance in 33% of reports.

• In 25% of reports, sample size was 20 patients per group or fewer.

The main concern with such "equivalence claims" is certainly the risk of harm to patients, as poor sensitivity has the potential to cause a type II error (false conclusion of no efficacy) and thereby to thwart satisfaction of public health needs for effective medicines.

Just as important as paying careful attention to all aspects of assay sensitivity is acknowledging from the outset that a large number of pharmaceutical products present sensitivity problems. That is, agents otherwise known to be clinically effective are often indistinguishable from placebo in well-designed and well-conducted trials. For this reason, such drugs are useless as comparators in active-controlled trials. A typical example is ondansetron, an antiemetic that, despite its known clinical effectiveness, showed no effect in many placebo-controlled trials [34]. Claims of equivalence of a new antiemetic agent with ondansetron would therefore be unreliable, given the lack of assay sensitivity of ondansetron (despite many trials in which it had proven to be superior in comparison with placebo). Similar examples include agents belonging to the class of antidepresssants, analgesics [35], beta-blockers used in postinfarct patients [36], antihypertensives, ACE inhibitors used in patients with heart failure, antianginal agents, and antihistamines.

The explanation for this serious problem lies in the great variability of the random placebo effect, which at times may profoundly confound the direction and the magnitude of treatment effects, especially in studies based on small sample sizes [37]. Regarding the example of ondansetron, the incidence of nausea and vomiting ranged from 10% to 96% in the placebo-controlled trials.

Furthermore, regarding situations in which multiple trials have demonstrated the efficacy of the active control when compared to placebo, the potential exists for referral bias due to eventual nonreporting of negative results. The risk in such instances is that the smallest clinically relevant effect of the control drug may not be valid [38].

## Rationale for choice of active control

In contrast to the scenarios described above, equivalence and noninferiority trials should be undertaken only when a well-proven standard therapy exists (i.e., when the intended control drug is accepted as the standard of care for the particular indication). Investigators should be confident that the efficacy of the control drug was proven to be superior in a previous placebo-controlled trial and that this efficacy will be preserved under the conditions of the current trial (i.e., the control drug has an established, predictable and quantifiable effect). Doubts about the validity of these assumptions mean uncertainty as to whether the two drugs in the current trial, which are allegedly equivalent, really are effective to a similar degree, or are equally ineffective, or cannot be evaluated definitively because the trial design was inadequate to demonstrate the real differences between the two agents.

## The goal of showing equivalence

A recent editorial by Alderson [39] concluded as follows: "We need to create a culture that is comfortable with estimating and discussing uncertainty." This observation applies especially to the field of equivalence/noninferiority trials. Increasing the degree of certainty in these trials is a matter of paying careful attention to the elements of study design, conduct, and analysis – all supposed to mirror as closely as possible the design, conduct, and analysis performed in previous evaluations of the current active control against placebo. Such trials should be reported in a transparent and explicit fashion, to acknowledge that they are not really equivalent to superiority trials [40].

The primary objective of equivalence/noninferiority trials is to demonstrate that the efficacy of the new treatment matches that of the control treatment. However, "equivalence" should not be interpreted to mean 100% (absolute equivalence can never be demonstrated), but that despite some degree of difference, the two agents are clinically indistinguishable. Closer scrutiny should be afforded the secondary objectives of the study, as they might demonstrate some sort of superiority over the control, such as a more favorable safety profile, easier administration, or reduced cost. Alternatively, results might indicate that the new agent would be a reliable second-line treatment.

All too often in the past, when trials that were designed to demonstrate the superiority of an agent over its comparator failed to reject the null hypothesis (i.e., a statistically significant difference was not demonstrated), results were interpreted as proof of the equivalence of the two drugs. A dangerous mismatch of the goals of the superiority and equivalence trials arises when the general reasoning employed in planning and evaluating superiority trials is simply extrapolated to active-controlled trials.

The aim of the superiority trial is to rule out the equality of the two agents being compared by rejecting the null hypothesis that the two agents are the same. Failure to reject the null hypothesis does not mean that equivalence can be assumed. Lack of superiority might be consistent with equivalence but does not prove it. In other words, "absence of evidence of a difference is not evidence of absence of a difference" [41].

In equivalence trials, the goal is to rule out all differences of clinical importance between the two agents being compared. This goal is accomplished by rejecting the null hypothesis that the smallest difference of clinical importance exists in favor of the standard-of-care regimen (i.e., in favor of the active control in the current trial). Therefore, establishing equivalence is contingent upon determining what specifically and precisely constitutes a clinically important difference. This process translates into the need to prove that the two interventions do not differ by more than a certain amount, defined as the "equivalence margin" (i.e., the tested agent is not inferior to the active control by more than the predefined margin).

## Methodological requirements

### Patient compliance with therapy

To assure the adequacy of the compliance component of assay sensitivity, prescreening of subjects selected to participate in active-controlled trials is necessary, as is reliable assessment of patient compliance with the trial requirements by means of appropriate methodologies [42,43]. Commonly, compliance is defined as the degree of correspondence between the patient's current dosing history and the prescribed drug regimen [44]. This seemingly simple definition covers the wide variability in patient compliance in the use of prescribed drugs. The degree of drug exposure has an impact on important clinical outcome variables and cost-effectiveness parameters [45,46]. Knowledge of the drug's kinetics and dynamics may allow pharmacokinetic/pharmacodynamic modeling, to address the consequences of temporal dosing patterns that result from variations in patient compliance with the recommended treatment regimen [17].

The most compelling example of treatment noncompliance occurs with antihypertensive medications. Noncompliance seems to be the main reason that blood pressure is adequately controlled in fewer than one fourth of patients treated for hypertension, both in the US and in European countries [47,48]. The classical "pill count" method of assessment grossly overestimates patient compliance, as self-reporting of medication use is highly skewed toward reports of excellent compliance [49] Over-reliance on inaccurate self-reports of compliance in research studies can result in misleading conclusions about both the efficacy of treatment and the dose-response relationships [48]. Electronic pill boxes that register the date and time of each access have become the "gold standard" and could be a valuable complement to conventional self-reporting of compliance [50,51]. Furthermore, compliance with the protocol-specified regimen can be improved by prescreening patients who are eligible for recruitment to active-controlled studies, with the aim of assessing the ability of individuals to comply with study-specific requirements.

### Concomitant medication

Use of co-medication during active-controlled studies, whether the result of self-medication or prescription medication, can distort the study's final results. Co-medication can interfere with response to the tested drug or the control drug, or it can influence the trial endpoints and lead to false-positive conclusions of equivalence. Use of non-trial medication is quite common in clinical trials in general and should be assessed and minimized, particularly in active-controlled studies.

### Patients' baseline characteristics and outcome features

A basic assumption is that the active agent in an equivalence/noninferiority study should have retained its known (historical) effect, demonstrated in a previous placebo-controlled comparison. Patients participating in the current study should be as similar as possible to the patients in the placebo-controlled trial with respect to all baseline values and treatment variables that might influence outcome. These variables include symptoms, signs, risk factors, morbidity, compliance with therapy, responsiveness to drug effects, nonuse of prohibited concomitant medication, consistent diagnostic criteria, inclusion and exclusion criteria, unbiased assessment of endpoints, and reasons for dropping out. Failure to achieve this similarity from the outset, failure to ensure high-quality study conduct, or both, can introduce bias into the study and compromise assay sensitivity.

The classical method to minimize systematic differences between study groups is *randomization*, (i.e., random allocation of patients to test or control groups). Further, *double blinding *is intended to minimize potential biases resulting from differences in management, treatment, or assessment of patients, or differences in interpretation of results that could arise as a result of the subject's or investigator's knowledge of the assigned treatment.

The type and frequency of outcome events in the current study are expected to be similar to those in the placebo-controlled comparison. Substantial differences, resulting most often from an imbalance in one or more of the variables mentioned above would render interpretation of differences between the new therapy and the active control very difficult. For example, because of lower baseline blood pressure values and fewer associated risk factors, patients in a hypertension study may display fewer outcome events.

### Choice and importance of outcome variables

Equivalence trials are commonly designed to demonstrate that the test treatment is similar in efficacy to the active control, the assumption being that the control treatment is effective under the conditions of the current trial. In reality, however, most equivalence trials are actually noninferiority trials, attempting to show that the new drug is not less effective than the control by more than the defined amount (margin). Presence of assay sensitivity is essential for interpretation of such a study. In cases with doubtful assay sensitivity, a three-arm study design (test drug, active control, and placebo) might be optimal. Apart from being more complex and requiring a larger sample size, such a trial offers the advantage of measuring the effect of the test drug versus placebo while allowing comparison of the test drug and active control in a setting in which assay sensitivity is established by the active control-versus-placebo comparison. By making the active groups in such trials larger than the placebo groups, it is possible to increase the precision of the active drug comparison while minimizing the chance that patients will be randomly assigned to placebo groups. Furthermore, this design allows distinction between adverse events due to the drug and those due to underlying disease ("background noise") [52].

As mentioned earlier, equivalence/noninferiority trials should not only focus on efficacy, but also should prospectively define an analytical plan for safety assessment as a secondary objective. Accordingly, appropriate statistical power to detect adverse effects is a necessity, as is collection of data on the comparative safety of each treatment. Failure to meet these requirements not only undermines the chance to exploit a potentially favorable safety profile of the test drug versus the control, but also presents the risk of missing dangerous signals with regard to safety. A recent example relates to mibefradil, a calcium inhibitor that appeared to have an excellent safety profile until postmarketing surveillance revealed cases of sudden death in patients at high risk for polymorphic ventricular tachycardia or patients in whom concomitantly administered drugs either inhibited mibefradil metabolism or otherwise amplified its cardiac risk [53]. A more recent experience with COX-2 inhibitors illustrates another significant problem in current evaluations of new drug safety. In the VIGOR study [54], the extensively marketed product rofecoxib appeared to be inferior to naproxen with regard to the frequency of cardiovascular thrombotic events, raising the question of rofecoxib's inferiority versus naproxen's superiority to an imputed placebo [55].

## Confidence interval and sample size

The margin (Δ) itself clearly communicates a judgment as to what is and is not important. The margin defines the largest difference that is clinically acceptable. Setting the margin is critical to the design of both equivalence and noninferiority trials, and it is commonly established for the purpose of excluding a clinically important difference between treatments. However, what constitutes such a difference may vary widely for each patient and clinician and might fall below the margins set by the designer of the trial. For that reason, careful clinical judgment and statistical reasoning should be exercised in selecting a meaningful difference to be ruled out; furthermore, this difference should be specified and justified a priori in all equivalence/noninferiority trials. At the very least, the equivalence margin should be smaller than the lower 95% confidence limit for the absolute risk difference observed between standard therapy and placebo in the relevant superiority trial [31]. That is, this lower boundary of the 95% confidence interval is the smallest expected effect of the control over the placebo, and it should exceed the established margin [29,38].

**Figure 1:**
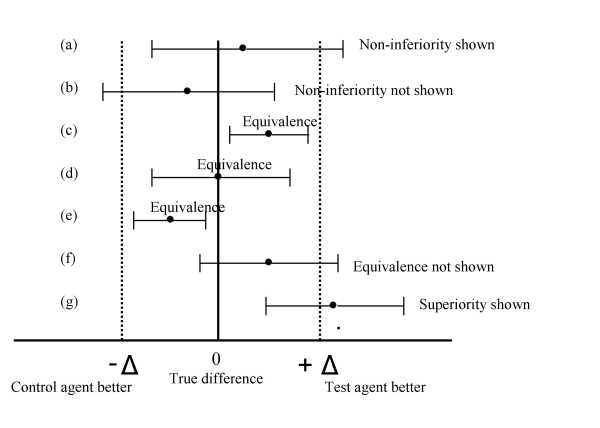
Confidence interval approach to analysis of equivalence and non-inferiority trials.

Confidence interval is the method of choice to interpret equivalence and noninferiority trials. It defines a range for the possible true differences between the test drug and the active control. If every point within this range reflects a difference that is clinically nonrelevant, then the two agents may be considered equivalent. In other words, for an equivalence trial, the two-sided 95% confidence interval – defining the range of possible differences between the test and the control agent – should lie entirely within the interval (-Δ to +Δ) (Fig. 1, lines c, d, and e).

For a noninferiority trial, the effect of the new drug may be shown to be similar to or greater than that of the control. The possible difference of interest occurs only in the - Δ direction, and the 95% confidence interval should lie entirely to the right of the - Δ value (Fig. 1, line a). A p value associated with the null hypothesis of noninferiority can be calculated.

A trial that is intended to demonstrate noninferiority may actually allow a claim of superiority for the test drug. In such a case, the one-sided 95% confidence interval lies to the right of not only the - Δ but also the zero line (Fig. 1, line g). A p value can be calculated to verify whether the superiority test is sufficiently small to reject the hypothesis of no difference at the 5% α level (p < 0.05). A claim of superiority, however, would imply a careful assessment of the test drug's safety profile, which should be similar to or better than that of the control to increase the strength of the evidence in favor of superiority. A less favorable safety profile raises the question of whether the claimed superiority outweighs the eventual adverse effects and therefore requires a careful quantification of the overall risk-benefit in clinical terms. This latter emphasis is meant as a reminder that claimed superiority of a test agent in the context of a noninferiority trial is, in fact, superiority to "no treatment," based on the proven superiority of the control agent against placebo in a previous trial.

Another possible scenario is that of a superiority trial that fails to detect a significant difference between the two agents being compared. An investigator who anticipates this outcome at the outset of the trial may want to downgrade the goal from superiority to noninferiority. That change is legitimate, provided that a noninferiority margin has been prospectively defined and the 95% confidence interval shown to lie to the right of the - Δ (Fig. 1, lines a and f). A post hoc definition of the margin is not acceptable.

For calculating sample size, values should be specified for the range of equivalence (Δ), and the α (type I error) and β (type II error) values should be selected on the basis of the same principles as for comparative trials. The distinction between one-sided and two-sided tests of statistical significance carries over into the confidence interval approach recommended by the Committee for Proprietary Medicine Products (CPMP) guidelines [56,57], which provide key information for making decisions about equivalence/noninferiority.

Unlike superiority trials, in which intention-to-treat analysis is the rule, in equivalence/noninferiority trials both intention-to-treat and per-protocol analysis should be run. Both types of analysis would be expected to lead to similar conclusions for a robust interpretation. In a recent article, Gomberg-Maitland et al. [58] suggested the use of standard guidelines for reporting equivalence/noninferiority trials to facilitate qualitative assessment of the methodology applied (Table 1).

**Table 1:** Guidelines for reporting equivalence/noninferiority trials. (*From Gomberg-Maitland et al*).

1. A table comparing the inclusion and exclusion criteria with those of previous trials on which the standard therapy was based.
2. A flow diagram delineating the number of eligible patients screened, the number randomised, the number of patients assigned to each group, the number of withdrawals and crossovers, and the number of patients in each group who successfully completed the trial on assigned treatment.
3. A statement on the projected and actual total treatment exposure (patient-years), the minimum per-patient exposure, and the respective impact of withdrawals and crossovers on exposure to initially assigned treatment.
4. The rationale of setting the margin of acceptable difference with specific reference to the minimum clinically important treatment effect and with the established efficacy advantage for the control over placebo. Where event rate ratios or floating margins are utilized, the rationale for their use, their prespecified criteria for adjustment, and the margin or ratios used to determine sample size should be provided.
5. The minimum requisite number of primary events should be established at the outset.
6. A comparison of event rates during treatment with the active control in the trial and in the historical trials that established its efficacy compared with placebo.

## Conclusions

Each year, major advances in drug discovery generate a seemingly endless supply of new drugs in virtually all therapeutic areas; however, in most cases these new drugs provide only incremental improvement in efficacy, safety, or the overall risk-benefit ratio. In addition, ethics-based restrictions on the use of placebos as comparators have enhanced the viability of equivalence and noninferiority trials as viable alternatives for registration purposes, for risk management once the drug is on the market, and for a marked increase in confidence in the new drug on the part of doctors and patients.

Given the complexity of these study designs, careful attention should be paid to the proper use of specific epidemiological features so as to avoid methodological deficiencies that may harm patients if clinically inferior treatments are erroneously deemed equivalent to the standard of care, or if potentially superior therapies are discarded as merely "equivalent."

## References

[B1] Hutton J, Borowitz M, Oleksy I, Luce BR (1994). The pharmaceutical industry and health reform: lessons from Europe. Health Aff (Millwood).

[B2] Williams A (1974). The cost-benefit approach. Br Med Bull.

[B3] Drummond MF, Stoddard GL, Torrance GW (1997). Methods for the economic evaluation of health care programmes.

[B4] Maynard A (1990). The design of future cost-benefit studies. Am Heart J.

[B5] Spurgeon D (2004). NIH promotes use of lower cost drugs for hypertension. BMJ.

[B6] (2002). Major outcomes in high-risk hypertensive patients randomized to angiotensin-converting enzyme inhibitor or calcium channel blocker vs diuretic: The Antihypertensive and Lipid-Lowering Treatment to Prevent Heart Attack Trial (ALLHAT). JAMA.

[B7] Dong BJ, Hauck WW, Gambertoglio JG, Gee L, White JR, Bubp JL, Greenspan FS (1997). Bioequivalence of generic and brand-name levothyroxine products in the treatment of hypothyroidism. JAMA.

[B8] Oles KS, Penry JK, Smith LD, Anderson RL, Dean JC, Riela AR (1992). Therapeutic bioequivalence study of brand name versus generic carbamazepine. Neurology.

[B9] Oberlin O, Leverger G, Pacquement H, Raquin MA, Chompret A, Habrand JL (1992). Low-dose radiation therapy and reduced chemotherapy in childhood Hodgkin's disease: the experience of the French Society of Pediatric Oncology. J Clin Oncol.

[B10] Lefebvre JL, Chevalier D, Luboinski B, Kirkpatrick A, Collette L, Sahmoud T (1996). Larynx preservation in pyriform sinus cancer: preliminary results of a European Organization for Research and Treatment of Cancer phase III trial. EORTC Head and Neck Cancer Cooperative Group. J Natl Cancer Inst.

[B11] Taggart SC, Custovic A, Richards DH, Woodcock A (1995). GR106642X: a new, non-ozone depleting propellant for inhalers. BMJ.

[B12] Edwards SJ, Lilford RJ, Braunholt DA, Jackson JC, Hewison J, Thornton J (1998). Ethical issues in the design and conduct of randomised controlled trials. Health Technol Assess.

[B13] Rothman KJ, Michels KB, Baum B (2000). Declaration of Helsinki should be strengthened: for and against. BMJ.

[B14] Mathe G, Brienza S (1988). From methodology to ethics and from ethics to methodology. Biomed Pharmacother.

[B15] Food and Drug Administration, Department of Health and Human Services (2001). Guidance for industry: E 10: choice of control group and related issues in clinical trials. Rockville, Md.

[B16] Ellenberg SS, Temple R (2000). Placebo-controlled trials and active-control-trials in the evaluation of new treatments. Part 2: practical issues and specific cases. Ann Intern Med.

[B17] Urquhart J (2001). Demonstrating effectiveness in post-placebo era. Clin Pharmacol Ther.

[B18] Rothman KJ, Michels KB (1994). The continuing unethical use of placebo controls. N Engl J Med.

[B19] Agostoni A (1995). Placebo and EU guidelines [Letter]. Lancet.

[B20] Aspinall RL, Goodman NW (1995). Denial of effective treatment and poor quality of clinical information in placebo controlled trials of ondansetron for postoperative nausea and vomiting: a review of published trials. BMJ.

[B21] Henry D, Hill S (1995). Comparing treatments [Editorial]. BMJ.

[B22] Freedman B, Weijer C, Glass KC (1996). Placebo orthodoxy in clinical research. I: Empirical and methodological myths. J Law Med Ethics.

[B23] Freedman B, Weijer C, Glass KC (1996). Placebo orthodoxy in clinical research. II: Ethical, legal, and regulatory myths. J Law Med Ethics.

[B24] Angell M (1997). The ethics of clinical research in the Third World [Editorial]. N Engl J Med.

[B25] Garattini S, Bertele V (2001). Adjusting Europe's drug regulations to public health needs. Lancet.

[B26] Jones B, Jarvis P, Lewis JA (1996). Trials to assess equivalence: the importance of rigorous methods. BMJ.

[B27] Ware JH, Antman EM (1997). Equivalence trials. N Engl J Med.

[B28] Ebbutt AF, Frith L (1998). Practical issues in equivalence trials. Stat Med.

[B29] Fleming TR (2000). Design and interpretation of equivalence trials. Am Heart J.

[B30] Snapinn SM (2000). Noninferiority trials. Curr Control Trials Cardiovasc.

[B31] McAlister FA, Sackett DL (2001). Active-control equivalence trials and antihypertensive agents. Am J Med.

[B32] Wang S-J, Hung HMJ, Tsong Y (2002). Utility and pitfalls of some statistical methods in active controlled clinical trials. Control Clin Trials.

[B33] Greene Wl, Concato J, Feinstein AR (2000). Claims of equivalence in medical research: are they supported by the evidence?. Ann Intern Med.

[B34] Tramer MR, Reynolds DJ, Moore RA, McQuay HJ (1998). When placebo controlled trials are essential and equivalence trials are inadequate. BMJ.

[B35] Max MB (1994). Divergent traditions in analgesic clinical trials. Clin Pharmacol Ther.

[B36] Yusuf S, Peto R, Lewis J, Collins R, Sleight P (1985). Beta blockade during and after myocardial infarction: an overview of the randomized trials. Prog Cardiovasc Dis.

[B37] Ernst E, Resch KL (1995). Concept of true and perceived placebo effects. BMJ.

[B38] Siegel JP (2000). Equivalence and noninferiority trials. Am Heart J.

[B39] Alderson P (2004). Absence of evidence is not evidence of absence. BMJ.

[B40] Djulbegovic B, Clarke M (2001). Scientific and ethical issues in equivalence trials. JAMA.

[B41] Altman DG, Bland JM (1995). Absence of evidence is not evidence of absence. BMJ.

[B42] Probstfield JL, Cramer JA, Spilker B (1991). The clinical trial prerandomization compliance (adherence) screen. Patient compliance in medical practice and clinical trials.

[B43] Knipschild P, Leffers P, Feinstein AR (1991). The qualification period. J Clin Epidemiol.

[B44] Urquhart J, de Klerk E (1998). Contending paradigms for the interpretation of data on patient compliance with therapeutic drug regimens. Stat Med.

[B45] Vrijens B, Geotghebeur E (1999). The impact of compliance in pharmacokinetic studies. Stat Methods Med Res.

[B46] Paterson DL, Swindells S, Mohr J, Brester M, Vergis EN, Squier C (2000). Adherence to protease inhibitor therapy and outcomes in patients with HIV infection. Ann Intern Med.

[B47] Burt VL, Whelton P, Roccella EJ, Brown C, Cutler JA, Higgins M (1995). Prevalence of hypertension in the US adult population. Results from the Third National Health and Nutrition Examination Survey, 1988–1991. Hypertension.

[B48] Walley T, Duggan AK, HAycox AR, Noziol CJ (2003). Treatment for newly diagnosed hypertension: patterns of prescribing and antihypertensive effectiveness in the UK. J R Soc Med.

[B49] Rand CS, Stone AA, Turkkan JS, Bachrach CA, Jobe JB, Kurtzman HS, Cain VS (2000). I took the medicine like you told me, doctor: self-report of adherence to medical regimens. In The science of self-report: implications for research and practice.

[B50] Vaur L, VAisse B, Genes N, Elkik F, Legrand C, Poggi L (1999). Use of electronic pill boxes to assess risk of poor treatment compliance: results of a large-scale trial. Am J Hypertens.

[B51] Burke LE, Burke LE, Ockene IS (2001). Electronic measurement. Compliance in healthcare and research.

[B52] Pledger G, Hall DB, Peace KE (1990). Active control equivalence studies: do they address the efficacy issue?. Statistical Issues in Drug Research and Development.

[B53] Glaser S, Steinbach M, Opitz C, Wruck U, Kleber FX (2001). Torsades de pointes caused by Mibefradil. Eur J Heart Fail.

[B54] Weir MR, Sperling RS, Reicin A, Gertz BJ (2003). Selective COX-2 inhibition and cardiovascular effects: a review of the rofecoxib development program. Am Heart J.

[B55] Konstam MA (2003). Matters of the heart: Assessing the cardiovascular safety of new drugs. Am Heart J.

[B56] (1995). Biostatistical methodology in clinical trials in applications for marketing authorizations for medicinal products. CPMP Working Party on Efficacy of Medicinal Products Note for guidance III/3630/92-EN. Stat Med.

[B57] Committee for Proprietary Medicinal Products (2001). Points to consider on switching between superiority and non-inferiority. Br J Clin Pharmacol.

[B58] Gomberg-Maitland M, Frison L, Halperin JL (2003). Active-control clinical trials to establish equivalence or noninferiority: methodological and statistical concepts linked to quality. Am Heart J.

